# Effects of Turning Frequency on Ammonia Emission during the Composting of Chicken Manure and Soybean Straw

**DOI:** 10.3390/molecules27020472

**Published:** 2022-01-12

**Authors:** Qianqian Ma, Yanli Li, Jianming Xue, Dengmiao Cheng, Zhaojun Li

**Affiliations:** 1 Key Laboratory of Plant Nutrition and Fertilizer, Ministry of Agriculture and Rural Affairs, Institute of Agricultural Resources and Regional Planning, Chinese Academy of Agricultural Sciences, Beijing 100081, China; mqq1027@126.com (Q.M.); liyanli02@caas.cn (Y.L.); 2 China-New Zealand Joint Laboratory for Soil Molecular Ecology, Institute of Agricultural Resources and Regional Planning, Chinese Academy of Agricultural Sciences, Beijing 100081, China; 3 SCION, Private Bag 29237, Christchurch 8440, New Zealand; jianming.xue@scionresearch.com; 4 College of Biology and the Environment, Nanjing Forestry University, Nanjing 210037, China; 5 Research Center for Eco-Environmental Engineering, Dongguan University of Technology, Dongguan 523808, China; chengdm@dgut.edu.cn

**Keywords:** composting, turning frequency, ammonia oxidizing bacterial, ammonia oxidizing archaeal, N fractions, ammonia emission

## Abstract

Here, we investigated the impact of different turning frequency (TF) on dynamic changes of N fractions, NH_3_ emission and bacterial/archaeal community during chicken manure composting. Compared to higher TF (i.e., turning every 1 or 3 days in CMS1 or CMS3 treatments, respectively), lower TF (i.e., turning every 5 or 7 days in CMS5 or CMS7 treatments, respectively) decreased NH_3_ emission by 11.42–18.95%. Compared with CMS1, CMS3 and CMS7 treatments, the total nitrogen loss of CMS5 decreased by 38.03%, 17.06% and 24.76%, respectively. Ammonia oxidizing bacterial/archaeal (AOB/AOA) communities analysis revealed that the relative abundance of *Nitrosospira* and *Nitrososphaera* was higher in lower TF treatment during the thermophilic and cooling stages, which could contribute to the reduction of NH_3_ emission. Thus, different TF had a great influence on NH_3_ emission and microbial community during composting. It is practically feasible to increase the abundance of AOB/AOA through adjusting TF and reduce NH_3_ emission the loss of nitrogen during chicken manure composting.

## 1. Introduction

As a sustainable, effective and ecofriendly approach to deal with livestock and poultry waste, composting is a dynamic biological process driven by microbial populations, self-heating and the biodegradative process of waste [1,2,3,4]. Composting manure has been shown to have a lot of agronomic benefits, such as a reduction in waste material mass and water content, pathogen suppression, weed seeds killing, and reduction of phytotoxic substances and unpleasant odors, eventually turning the manure into a stable nutrient source of organic fertilizer needed for crop production [5,6]. However, the large amount of loss of nitrogen during the composting process is one of the key disadvantages of conventional aerobic composting [6,7,8]. Studies have shown that about 16–74% of the total nitrogen (TN) at the initial stage is lost and approximately 46.8–77.4% of the TN is unavoidably lost with the release of NH_3_ during composting [9,10]. The NH_3_ emissions can not only result of reducing the quality of compost products but also cause secondary environmental pollution [11,12,13]. Therefore, it is necessary to lower the emission of NH_3_ during composting in order to minimize environmental impacts. 

The differences in transfer rate of heat as well as mass can cause spatial gradient of air humidity, oxygen content, temperature, volatile solid content and so on. These can further lead to less porosity and poor ventilation in the compost [14]. The aforementioned physicochemical properties of the pile including temperature, humidity and porosity could influence nitrogen loss and NH_3_ emission, but also the quality of compost products during aerobic composting [12,15]. It has been shown that turning of composts can aerate the composting pile by increasing porosity, further promoting activities of microbial organisms that are responsible for degrading the compost materials and generating the heat. Therefore, turning is needed to achieve a homogeneous fermentation process [16] and ensure the abundant oxygen of the compost [17]. Previous research demonstrated that there was a strong relationship between turning frequency (TF) and some physicochemical indicators of compost or compost maturity [18,19]. For instance, the TF could affect total bacterial abundance, TN, temperature, pH, content of moisture, ratio of C/N and germination index (GI) of composting piles [19,20,21]. Aeration also strongly relates to composting efficiency and gas emission in the aerobic fermentation process. It seemed that turning of materials is the most convenient and widely used method of aeration for the minimization of TN loss and NH_3_ emissions during composting, which can promote the fermentation process [19,22]. 

So far, many studies have shown that TF can improve the quality of compost [19,22,23]. For example, proper TF (every five days) could improve final product quality (pathogen reduction) and cut down the composting time of garden waste (30~36 days) [24]. In addition, the longest thermophilic stage were obtained with TF of every 7 days compared to every 5, 10 and 15 days when composting Camellia oleifera shell with goat manure [23]. Soto-Paz et al. also found that it took less time to reach the maximum temperature with higher TF when he investigated the effects of TF (1, 2 and 3 turnings/week) on co-composting of biowaste and sugarcane filter-cake. It is thought that the increasing TF may reduce compaction and ensure aeration inside piles, which further results in higher biological activity and consequently larger heat release [25]. However, higher frequency of turning may cause more depletion of degradable materials attributed to a higher heat, water loss and NH_3_ emission through evaporation and convection. Turning the compost material more frequently from once weekly to daily could cause more loss of TN, but this varied with different cases [25]. Both excessive and less turning could significantly affect the decomposition of piles through reducing material temperature and humidity, which reduces the loss of nutrients, results in long fermentation period and bad quality of the final product [19,26]. Therefore, choosing appropriate TF is very important for achieving an effective composting process.

The nitrogen loss mainly in NH_3_ emission is the most prominent factor limiting the efficient use of manure [27]. The nitrate formation from NH_3_ oxidation has been considered as a way to preserve nitrogen in the final product of composting [9]. The conversion of ammonia to nitrate (i.e., nitrification) during the assimilation process is inseparable from the ammonia-oxidizing bacteria or archaea (AOB/AOA) [9]. In recent years, the increasing and widespread attention were paid on impacts of microbial composition on the quality and maturation of compost [28,29]. Oxidation of ammonia driven by AOB and AOA producing ammonia monooxygenase (*amo*A) is the first and rate-limiting step of nitrification [9]. In general, organic matter is decomposed during the composting process consisted of complex bioprocessing, relying on the activity of microorganisms. It is crucial for successful composting to achieve and maintain a favorable composition of microorganisms [30]. Therefore, exploring the dynamic changes of key functional groups (e.g., AOB, AOA) of microbial communities in relation to NH_3_ emissions under different turning frequencies of the composting materials will provide better insight into the AOB and AOA regulations of NH_3_ emissions for minimizing the nitrogen losses during composting. 

In our study, we analyzed the structure and diversity of bacterial communities, also including AOB and AOA abundance to quantify the target microbes responsible for nitrification by high-throughput sequencing of 16S rRNA gene amplification. The main objectives of this study were: (1) to understand the effects of TF on composting and product quality, N transformation and NH_3_ emissions, and succession microbial structure and diversity during the composting, (2) to investigate if the contribution of AOB and AOA to ammonia oxidation could vary at different composting stages and be manipulated by TF, (3) to identify key driving factors shaping AOB and AOA communities and influencing NH_3_ emission and nitrogen transformation during the composting.

## 2. Materials and Methods

### 2.1. Composting Process and Sampling

The composting trial was conducted in an organic fertilizer factory located at Daxing District in Beijing, China. Composting materials applied in this experiment consisted of chicken manure (CM) and soybean straw (S). The fresh chicken manure was gathered from a large farm in Daxing District and was air dried (15–20 °C) reaching a water content of <30%, ground and sieved using a 10 mm mesh. The soybean straw came from farms located near the factory also air dried to a water content <10%, ground to a granule size smaller than 10 mm. The chicken manure and soybean straw were mixed well to get the compost with the initial C:N of 8:1, humidity of 60% [5].

Sixty kilograms of the above mixture were put into each of twelve plastic boxes (150 L) with lids. There were four treatments assigned with four different turning frequencies of the compost: (1) once a day (CMS1), (2) once every 3 days (CMS3), (3) once every 5 days (CMS5) and (4) once every 7 days (CMS7). Each TF treatment was repeated three times, with 12 boxes in total. The turning process of compost was carried out by mixing the treated substrate manually with a garden shovel. 

The 12 boxes with composting materials were arranged in a room according to the randomized block design. The temperature of composting piles was monitored twice a day (9:00 am and 18:00 pm) for calculating an average daily temperature. The duration of composting was 66 days and the pile temperature was close to room temperature in the end. The samples were collected after completely mixed on day 1, 3, 5, 7, 10, 12, 15, 17, 22, 29, 35, 43 and 66 during composting. To collect the representative sample from each replication box, five sub-samples were taken at five locations (one in the center and four at the corners) of a box at the depth of 15 cm, bulked together and mixed well, then kept in polyethylene bags. Each sample was split into three parts. The first part was air-dried for the dry-based chemical analysis. The second part was kept fresh at 4 °C to analyze NH_4_^+^-N, NO_3_^−^-N, etc., and the third was preserved at −80 °C for DNA extraction and 16S rRNA, bacterial *amo*A (AOB) and archaeal *amo*A (AOA) gene sequencing analyses.

### 2.2. Physicochemical Properties Analyses of NH_3_ Emission

All collected samples were analyzed for moisture content, pH, germination index (GI), TN and total carbon (TC), ammonium nitrogen (NH_4_^+^-N), nitrate nitrogen (NO_3_^−^-N). The moisture content was measured by drying the sample at 105 °C until constant weight was achieved. The fresh samples were used to measure pH, GI, NH_4_^+^-N and NO_3_^−^-N according to Test Methods for the Examination of Composting and Compost (TMECC, 2002). pH values were detected by MP521 pH meter (Shanghai, China) after extracted in 1:5 (*w*/*v*). The contents of TN and TC were measured for air-dried samples using elemental analyzer (Vario EL III, Elementar Analysensysteme GmbH, Langenselbold, Germany). To determine the concentration of NH_4_^+^-N and NO_3_^−^-N, fresh samples were extracted at 25 °C using 0.5 M K_2_SO_4_ (1:10 *w*/*v*), and the filtrates were analyzed by using indophenol blue technique [31,32]. Released ammonia was collected with the gas collecting device during composting processes. The NH_3_ emission was measured by adsorbing the exhaust gas with H_3_BO_3_ and titrated against HCl [33,34].

### 2.3. DNA Extraction, PCR Amplification and Sequence Analysis

DNA extraction was performed using the Fast DNA SPIN extraction kits (MP Biomedicals, Santa Ana, CA, USA) and subsequently kept at −80 °C for further analysis. The quantity of extracted DNA was determined using NanoDrop ND-1000 spectrophotometer (Thermo Fisher Scientific, Waltham, MA, USA) and the quality using agarose gel electrophoresis.

Bacterial 16S rRNA gene V3–V4 regions (total bacteria) was amplified using the forward primer 338F (5′-ACTCCTACGGGAGGCAGCA-3′) and the reverse primer 806R (5′-GGACTACH VGGGTWTCTAAT-3′). The specific primer sets for bacterial *amo*A (AOB) and archaeal *amo*A (AOA) gene were F(5′-GGGGTTTCTACTGGTGGT-3′), R(5′-CCCCTCKGSAAAGCCTTCTTC-3′) [35] and Arch-amoA26F (5′-GACTACATMTTCTAYACWGAYTGGGC-3′), Arch-*amo*A417R (5′-GGKGTC ATRTATGGWGGYAAYGTTGG-3′) [36], respectively. In present study, the related high-throughput sequencing analysis was processed using the Illumina MiSeq platform by Shanghai Personal Biotechnology Corporation., Ltd. (Shanghai, China) to have a closer look at the microbial communities.

Sequences were analyzed and quality-filtered using QIIME 2.0 (Quantitative Insights Into Microbial Ecology, Version 2019.4) and Vsearch (v2.13.4) software as described by [37,38]. In a word, raw sequence data were demultiplexed, quality filtered, denoised, merged and chimera removed using the DADA2, including a strict quality control by getting rid of reads with ambiguous bases, singletons and chimera [39]. The reads were selected to amplicon sequence variants (ASV), or unique sequences, using DADA2 and taxonomically identified. ASV taxonomic was classified through BLAST searching the representative sequences set against the Silva 132 [40] or NCBI Database [41]. 

### 2.4. Bioinformatics and Statistical Analysis

Microbial functions were analyzed by PICRUSt2 (Gavin M. Douglas, et al., preprint) upon MetaCyc (https://metacyc.org/ (accessed on 17 July 2021)) and KEGG (https://www.kegg.jp/ (accessed on 23 August 2021)) databases. Bioinformatic analyses of sequence data were mainly performed using QIIME 2.0. ASV-level alpha-diversity for microbial community was evaluated with Good’s coverage, Chao1, Observed species, Shannon index and Simpson index. Beta diversity analysis was carried out using Bray-Curtis and visualized by non-metric multidimensional scaling (NMDS) to investigate the structural variation and community function of microbial communities. Taxa relative abundances at both phylum and genus levels were statistically compared among treatments and visualized as bar chart. Linear discriminant analysis effect size (LEfSe) was conducted to test differentially abundant taxa between treatments using default parameters [42]. Random forest analysis was applied using “Random Forest” package in the R package [43] to discriminate the samples from various groups. The generalization error was estimated using ten-fold cross-validation for all comparisons from the 16S rRNA, bacterial *amo*A (AOB) and archaeal *amo*A (AOA) data. Microbial functions were predicted by PICRUSt 2, through high-quality sequences of 16S rRNA gene [44] based on MetaCyc (https://metacyc.org/ (accessed on 25 July 2021)).

One-way ANOVA was performed using SPSS19.0 software for testing the treatment effect. Duncan’s multiple range test was carried out to compare the significance levels between the means. The relationships between physicochemical parameters and total/ammonia-oxidizing bacterial/archaea community were assessed using Canoco 4.5. Spearman correlation analysis was also performed using SPSS19.0 software to obtain whether there was a significant correlation between environmental factors and species community. All figures for physicochemical parameters were plotted by Graphpad Prism 5.0.

## 3. Results and Discussion

### 3.1. Effect of TF on Composting Process, pH and GI Values of the Compost

**Figure 1:**
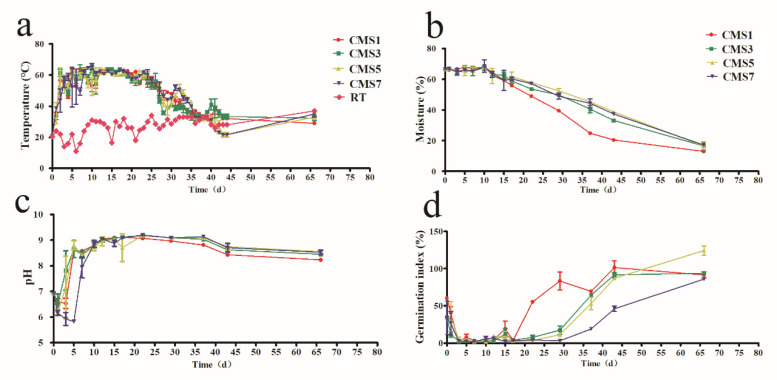
Changes in temperature (**a**), moisture (**b**), pH (**c**) and germination index (**d**) at different treatments during the composting. Every data point represents the mean of three replicates. Bars indicate the SDs of the means.

In this study, the whole composting process could be divided into four phases, namely, mesophilic phase (21–40 °C, days 0–1), thermophilic phase (35–65 °C, days 2–17), cooling phase (65–40 °C, days 18–35) and maturation phase (40–25 °C, days 36–43) according to the temperature variation trend of composting. These are consistent with other researcher’s results [45]. For all TF treatments, the temperature rose rapidly at the beginning and entered the thermophilic phase (>55 °C) on day 2 (Figure 1a). In the present study, the pile temperature at the CMS7 treatment was kept above 60 °C for a total of 11 days, which was three days less than the other three treatments. In addition, CMS1 treatment remained above 55 °C for 18 days, with 3 days less than that of CMS5. These results were in accordance with the previous studies. It has been shown that premature turning delayed time to achieve high temperatures and turning too late was not helpful for the temperature rising again and the high temperature keeping during composting [25,46]. Notably, such high TF aeration surplus as CMS1 treatment not only increased the costs of energy but also decreased the temperature of the composting pile quickly, hindering the thermophilic phase achievement [47]. However, too low TF (i.e., CMS7 treatment) resulted in lots of anaerobic zone present in the compost system and therefore caused problems of long fermentation time [48]. An increase in temperature was observed for all treatment after each compost turning as also reported by previous study [49], thereby indicating that proper TF may increase the oxygen content in the compost, which may result in promoting the activities of microbial organism that can degrade the materials in the compost pile and generating heat [17].

Moisture content can greatly impact the microbial activity and physicochemical parameters of the compost. As shown in Figure 1b. the moisture content of each treatment decreased from 65–66% at the beginning to 20.41%, 33.16%, 38.71% and 37.35% on day 43 for CMS1, CMS3, CMS5 and CMS7 treatments, respectively. The composting process may be hindered at higher TF, which might cause higher loss of compost moisture. It has been reported that moisture content is the strongest correlative factor with the succession of bacteria and archaea among numerous other factors such as pH, salinity and nutrients [50]. Therefore, TF should be appropriately regulated to obtain an efficient composting process.

A key factor which influences gaseous emissions as well as the microbial activities and structure during composting is pH [33,51]. In the present study, the overall patterns of pH changes during the composting period were similar for all treatments, which increased dramatically from 6.16–6.48 on day 1 to 8.86–9.17 on day 15 and then gradually increased to 9.23 on day 22 and afterwards reduced slightly (Figure 1c). The respiration microorganism and of ammonia (NH_3_) emission have significant impacts to the pH changes, as have been reported previously [52,53]. The lower pH obtained under CMS1 treatment during cooling and mature stage (*p* < 0.05), possibly resulted from the release of H^+^ ions in the process of nitrification during the transformation of organic nitrogen [54]. At the end of composting, the final pH of materials was also affected by ammonia volatilization [6]. 

The GI always increases with toxic materials degradation in composting pile [55]. As the composting process progressed, the GI values of CMS1, CMS3 and CMS5 increased, reaching to 91.6%, 93.77% and 124.36% at the end of composting time, respectively (Figure 1d). The compost is recognized basically and sufficiently mature when the value of GI is above 50% and enough well matured when GI value achieves 80% [56,57]. Thus, it can be concluded that the composts of CMS1, CMS3, and CMS5 were stabilized enough at day 43 but the longer time (than 43 days) was required for CMS7 to reach stabilization. TF is commonly thought to be a key factor affecting the rate of composting as well as compost quality [24]. Previous researchers have suggested that compared to every 4-day and every 7-day turning, every 2-day turning can facilitate faster sterilization and maturity during composting [22,58]. In this study, the GI values of the CMS1, CMS3 and CMS5 treatments were 101.52%, 91.64% and 87.24%, respectively, which were significantly higher than that of the CMS7 (46.19%, *p* < 0.05). 

### 3.2. Effect of TF on Nitrogen Transformation and NH_3_ Emission

**Figure 2:**
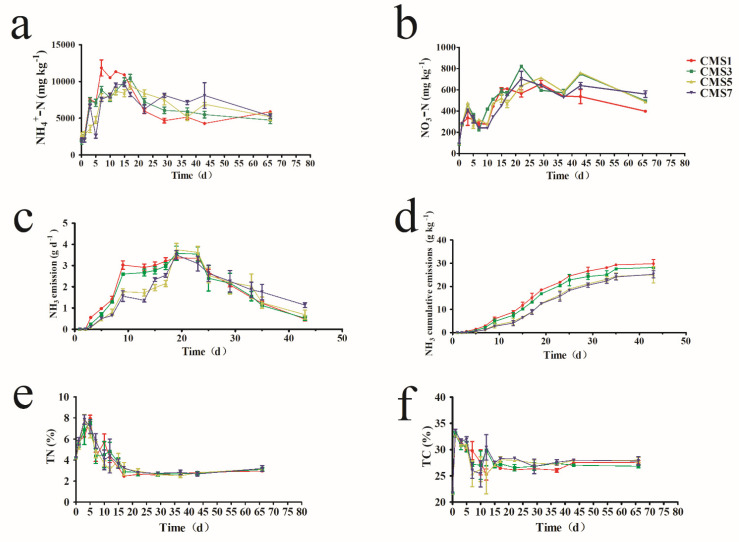
Variation in ammonium nitrogen (**a**), nitrate nitrogen (**b**), NH_3_ emission rate (**c**), cumulative NH_3_ emission (**d**), TN (**e**) and TC (**f**) in different treatments during the composting. Every data point represents the mean of three replicates. Bars indicate the SDs of the means.

As shown in Figure 2a, the concentration of NH_4_^+^-N showed an increasing trend among the four treatments with the temperature reaching its highest on day three. These were primarily attributed to organic nitrogen mineralization and ammonification [59]. The contents of NH_4_^+^-N in CMS1 and CMS3 group were significantly higher than that in CMS5 and CMS7 on day five suggested higher frequency and premature turning at the beginning of the composting process may promote rapid mineralization and the ammonification during composting (*p* < 0.05) [60]. The difference among the four treatments may be due to the combined effects of the degree of NH_3_ emissions, organic nitrogen hydrolysis, and nitrification during the composting [9]. During the composting process, NH_4_^+^-N might be transformed into NO_3_^−^-N by AOB and AOA, which could then result in reduction of NH_3_ emission, and decreasing the loss of organic nitrogen [61,62,63,64]. However, during thermophilic phase, the excessive NH_3_, high temperature and oxygen-deficient of pile can inhibit the proliferation and activity of nitrifying microbial communities [65]. Comprehensively speaking, losses of NH_4_^+^-N might be the result of the volatilization and nitrification process.

As shown in Figure 2b, the concentration of NO_3_^−^-N was low at 89.60–96.61 mg/kg initially then it increased to 263.63–483.12 mg/kg on day three during the early composting stages. The NO_3_^−^-N concentration decreased gradually during the cooling phase due to a reduction in the concentration of ammonium [66]. The NO_3_^−^-N content of CMS1 is significantly lower than that of CMS3, CMS5 or CMS7 during the cooling and maturity period (*p* < 0.05). This may because that most of the ammonium in CMS1 used as the substrate for generating NO_3_^−^-N was volatilized in the form of NH_3_ due to the high TF, reducing the substrate for ammonia-oxidation reaction for the nitrifying microorganisms [67]. Or the moisture content in the compost material was greatly reduced for CMS1, which inhibits the activity of nitrifying microorganisms [50]. In addition, compared with CMS1, CMS3 and CMS7 treatment, the TN loss of CMS5 was decreased by 38.03%, 17.06% and 24.76%, respectively (Figure 2e). However, because nitrogen metabolism is a complex process, synergistic carbon and nitrogen metabolism, for reducing the nitrogen losses, the activity and quantity of enzymes and the genes, should be further assessed during composting. The relatively appropriate TF may reduce the NH_3_ emissions through accelerating the proliferation of AOB and AOA. It was also testified by the changes of NH_4_^+^-N and NO_3_-N contents during composting.

As shown in Figure 2c, NH_3_ was detected at day 2. With the temperature and pH increased, NH_3_ emissions from all of the treatments rapidly increased and reached their highest values. The NH_3_ emission from the CMS1 treatment observed on the third, fifth, ninth and thirteenth day was significantly higher than the other three treatments (*p* < 0.05). The high rate of NH_3_ emissions for all treatments might be due to quick degradation of organic matter and the fast conversion of NH_4_^+^-N to NH_3_ [13,34,68], associated with the increased temperature and pH during the thermophilic stage [59,69]. Previous study also suggested the NH_3_ volatilization had a positive relationship with the amount of aeration or TF [70]. The characteristics of NH_3_ emissions from this study are in line with previous research [71,72]. Overall, physicochemical properties are important factors to influence NH_3_ volatilization such as temperature, pH, NH_4_^+^-N concentration, and the microbial community [12,73,74,75]. 

As shown in Figure 2d, the cumulative NH_3_ emission profiles indicated that more than 73.67% of NH_3_ emissions occurred during the first 23 days in treatments CMS1. However, in the same 23 days, less than 70% of the NH_3_ was emitted from the CMS 5, CMS 7 group while more than 70% and less than 73% for CMS3 group. The emission of NH_3_ was strongly connected with the temperature of the pile and microorganisms [74,76]. NH_3_ is mainly important gas causing nitrogen loss during composting. NH_3_ emission is mainly affected by temperature, pH, NH_4_^+^-N concentration, aeration rate, and moisture content [77]. The differences of NH_3_ emissions in all groups were probably due to the interrelationships between pH, temperature, aeration rate and moisture content [78]. The results showed that the higher TF might lead to the emission of relatively larger amount of accumulative NH_3_ emission during the early composting stage, but the detected concentration was similar between different TF groups during the late composting period. Therefore, in general, too high TF results in more ammonia emission during composting.

Figure 2e,f showed the variation of TN and TC content with composting time. TN contents for all treatments presented a similar trend. Compared with CMS1, CMS3 and CMS7 treatments, the TN loss of CMS5 decreased by 38.03%, 17.06% and 24.76%, respectively. Many studies have shown that 16~74% of the initial TN is lost during composting [75]. The decrease in TN might be due to the large amount of nitrogen loss caused by the NH_3_ volatilization, the degradation and mineralization of complex organic compounds [79]. Nitrogen fixing bacteria might also have contributed to a lesser degree to the increase in TN in the later phase of composting. The TC content of the compost gradually decreased with composting time. This may be attributed to the microorganisms mineralized the organic carbon as a source of energy. The C/N ratio data of compost materials and samples, as shown in the supplementary Table S6 to give more information about the type of composting waste. The ratio of C/N showed a decreasing trend among the four treatments at the first five days and increased on day 7.

### 3.3. Effects of TF on Bacterial Community Diversity and Composition during Composting

**Figure 3:**
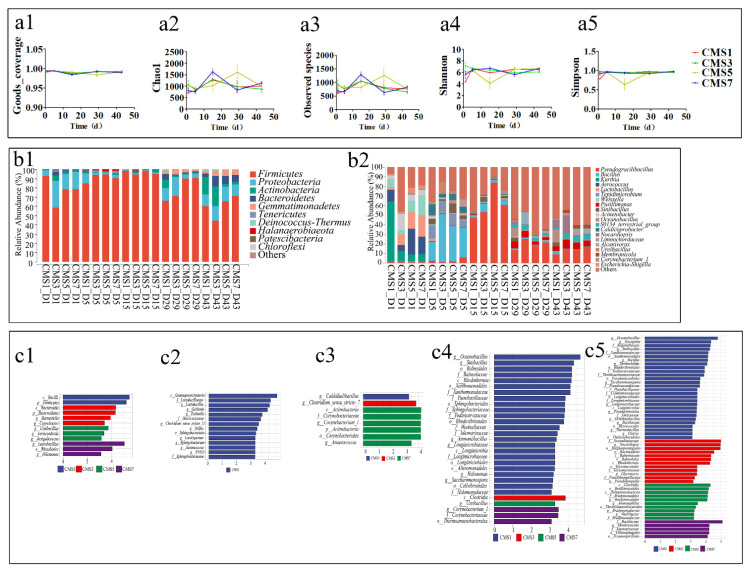
Alterations of bacterial community structure in different treatments during the composting. The α diversity of bacterial community in different treatments during composting process (**a1**–**a5**). The relative abundance of different bacteria at the (**b1**) phylum and (**b2**) genus levels in different treatments during the composting. LDA (Linear discriminant analysis) Effect Size (Lefse) analysis of the biomarkers ((**c1**–**c5**) represent day1, 5, 15, 29 and 43, respectively), showing biomarkers of the significant and biological differences from the phylum level to the genus level (LDA score > 3, *p* < 0.05).

The five alpha diversity indices (a1–a5) for each treatment at day 1, 5, 15, 29 and 43 during composting are shown in Figure 3(a1–a5), respectively. The Good’s_coverage index for all samples was over 0.99, indicated that the sequencing depth was enough for this bacterial community analysis. The microbial taxa abundance indices Chao and Observed species were significantly (*p* < 0.05) lower in the CMS5 treatment than other treatments at post-thermophilic stage (i.e., day 15), but higher at cooling stage (i.e., day 29). This suggested a significant effect of different TF on bacterial abundance, especially for the post-thermophilic and cooling stage. The microbial richness and evenness indices Shannon and Simpson were also significantly lower in the CMS5 treatment than other treatments at post-thermophilic stage (i.e., day 15). This indicates a selective effect of different TF against bacterial taxa at different composting stages. The Chao and Observed Species indices rose sharply at post-thermophilic stage of composting, which was attributed to the growth of a range of microbiome. Certain bacteria could proliferate during the thermophilic phase, such as amylolytic microorganisms as previous studies reported [80]. Previous research also suggested higher bacterial abundance and diversity during thermophilic stage of composting for green wastes [81]. However, research has also shown that microbial activities could be inhibited during thermophilic phase, and the diversity fall may be attributed to the dominance of some microorganism taxa [82].

The microbial diversity and phylogenetic distribution might have close relationship with the composting process and the quality of compost. Therefore, the composition and succession of bacterial communities at different stages were analyzed. The composition and relative abundance of bacterial communities at phylum and genus levels are shown in Figure 3(b1,b2), respectively. In total, we detected 27 phyla during composting, with Firmicutes, Proteobacteria, Actinobacteria, Bacteroidetes as the top 4 dominant bacteria, which accounted for 92.32–99.82% of the total sequencing reads. This finding agreed with previous studies [83,84]. These four bacterial phyla are prevalent throughout the whole composting period and have a strong ability to degradation of organic matter [85,86]. Firmicutes often show high enrichment throughout composting because of the ability to form endospores that can help them to keep surviving high temperatures and harsh environment [85,87,88,89]. In present study, Firmicutes also played a dominant role in the whole composting process, accounting for 44.24–98.20% of the relative abundance. Previous studies also suggested that Proteobacteria are typically the most (or second most) abundant phylum during most aerobic composting [84]. Interestingly, the relative abundance of Firmicutes in high TF treatments (i.e., CMS1 and CMS3) was significantly lower than that in other treatments, but Proteobacteria and Actinobacteria were significantly higher than that in other treatments during cooling and maturation stages. These results might explain a selective effect of higher TF against bacterial taxa during different composting stages and indicate high aeration conditions could stimulate the growth of aerobic bacteria such as Proteobacteria and Actinobacteria [90] but inhibit the activity of anaerobic microorganisms in Firmicutes [91]. Interestingly, the highest abundance of Gemmatimonadetes was found in the CMS5 treatment at the maturation stage. Previous studies also showed that Gemmatimonadetes was probiotics [92], significantly enriched at the maturation stage of vermicomposting with coconut leaf [93], and was predominant in soils amended with alkaline treatments [94]. Thus the beneficial microorganisms may be stimulated by appropriate TF or aeration during aerobic composting [95].

In total, we detected 569 genera during composting, with Pseudogracilibacillus, Bacillus, Kurthia, Aerococcus, Lactobacillus, Tepidimicrobium, Weissella, Pusillimonas, Sinibacillus and Acinetobacterwere as the top 10 dominant bacteria, which accounted for 27.57–91.19% of the total sequencing reads. These bacterial genera belong to the phyla of Firmicutes, Proteobacteria, Actinobacteria, Gemmatimonadetes and Bacteroidetes, respectively. Pseudogracilibacillus was the most dominant genus accounting for 1.01–83.53% at whole composting stages. The highest relative abundance of Pseudogracilibacillus was found at day 15 (47.27–83.53%), followed by the cooling and the maturation stages (13.52–25.12%). The ecological function of Pseudogracilibacillus during aerobic composting has seldom been reported. Previous study suggested that Pseudogracilibacillus as neutrophilic aerobes could exist in the high-temperature environments and be associated with the nitrogen cycle [96]. The second dominant genus was Bacillus, accounting for 0.35–50.06% at different composting stages. The relative abundance of Bacillus at the pre-thermophilic stage was 20.01–50.06%, which was significantly higher than that at other stages. This result was in agreement with previous studies, which have reported that the genus Bacillus consists of a large quantity of thermophilic bacteria and can dissimilate and reduce nitrogen compounds [97]. The relative abundance of Kurthia, Aerococcus, Lactobacillus, Weissella, and Acinetobacter accounted for higher than 1% only at the mesophilic phase (day 1). Pusillimonas has been previously identified as the main dominant bacterial community correlated with the heterotrophic nitrification and denitrification of composting and wastewater [98,99]. The abundance of Bacillus, Sinibacillus, Oceanobacillus and Nocardiopsis was significantly higher in CMS1 and CMS3 treatments than other treatments at cooling and mature stages (*p* < 0.05). On the contrary, the abundance of Pseudogracilibacillus, Pusillimonas, S0134_terrestrial_group, Limnochordaceae, Alcanivorax and Membranicola was significantly higher in CMS5 and CMS7 treatments at the maturation stage (*p* < 0.05). This suggested that these bacterial genera might be sensitive to aeration conditions and could be manipulated by TF of composting materials. Sinibacillus, Limnochordaceae and Oceanobacillus genera have been previously identified as the dominant communities responsible for the proteins transportation-related genus of composting [33,100] and wastewater [101]. Interestingly, as a facultative anaerobe with urease activity, the relative abundance of Pseudograciibacillus decreased with decreasing temperature, which may be attributed to the reduction in ammonia emission [102,103]. 

The significant differences among TF treatments for bacterial communities were identified by LEfSe (Figure 3(c1–c5), Supplementary Figure S2(a1–a5), Table S1). Compared to the mesophilic and thermophilic phases, more taxa were significantly affected by TF during cooling phase and maturation phase. The LEfSe of all taxa showed 12, 14, 8, 29 and 55 bacterial taxa had significant differences (LDA > 3, *p* < 0.05) among the treatments at mesophilic, pre-thermophilic, post-thermophilic, cooling and maturation phases, respectively. The dominant taxa (LDA > 4, *p* < 0.05) were phyla as Firmicutes, Bacteroidetes and genus as *Lactobacillus*, *Ureibacillus* during mesophilic phase, genus as *Lactobacillus* during pre-thermophilic phase, phyla as Actinobacteria and genus as *Corynebacterium_1* during post-thermophilic phase, genus as *Oceanobacillus* and *Snibacillus* during cooling phase, phyla as Bacteroidetes and genus as *Oceanobacillus*, *Georgenia*, *Sinibacillus*, *Bacillus*, *Thermobifida*, *Nocardiopsis, Bradymonadales* and Membranicola during maturation phase. It must be noted that microbial diversity is related to the physicochemical properties of compost, which change with the composting time [104]. LEfSe is an accurate and effective method to identify specific microbes (biomarkers) that displayed significant differences in microbial abundance between different treatments [105]. Among all the different taxas above, phyla as Firmicutes, Bacteroidetes, Actinobacteria and only genus as *Ureibacillus, Lactobacillus, Oceanobacillus, Sinibacillus, Corynebacterium_1, Membranicola* also belonged to the top relative abundance10 phylum and top 20 genus, respectively. This indicated that the dominant taxa were significantly affected by different TF. In addition, it should be noticed that the genus *Ureibacillus* was significantly affected by different TF during both mesophilic and cooling phase. The genus *Lactobacillus* was significantly affected by different TF during both mesophilic and pre-thermophilic phase [106]. Interestingly, *Sinibacillus, Corynebacterium_1* and *Oceanobacillus* were all significantly affected by TF for both post-thermophilic and cooling phase. Meanwhile, previous studies emphasized the importance of low-abundance microorganisms to ecosystem function, such as biochemical processes [107], community succession [108] and microbiome function [109]. Therefore, more attention to these different species caused by different TF during different stages may provide a certain amount of theoretical support for optimizing the composting process. 

The keystone taxa for the microbial communities of the four groups during composting were determined using a random forest model (Supplementary Figure S3(a1,a2)). At the phylum level, nine taxa were the dominant species occupying the top 10 abundance among the top 10 different important phylum for 16S rRNA while *Cyanobacteria* as less abundant species was out of the top 10 phylum in abundance. At the genus level, nine taxa were the dominant taxa occupying the top 20 different important genus for 16S rRNA, such as *Pseudogracilibacillu*s, *Caldicoprobacter, Aerococcus, S0134_terrestrial_group*, *Pusillimonas*, *Bacillus*, *Membranicola, Weissella, Ureibacillus* and *Alcanivorax.* It was also shown that these classes were common for dominating the composting process [110]. *Pusillimonas* was widely distributed in environments and can utilize a variety of fatty acids and urea [111]. *Pseudogracilibacillus*, *Ureibacillus* and *Alcanivorax* are affected by the oxygen concentration and related to nitrogen transformation [103,112,113,114]. So, it was suggested different TF could affect bacteria involved in N cycle, especially ammonium oxidation. Furthermore, TF can also alter the structure of the bacterial community.

### 3.4. Effects of TF on Ammonia-Oxidizing Bacteria/Ammonia-Oxidizing Archaeal Diversity and Composition during Composting

**Figure 4:**
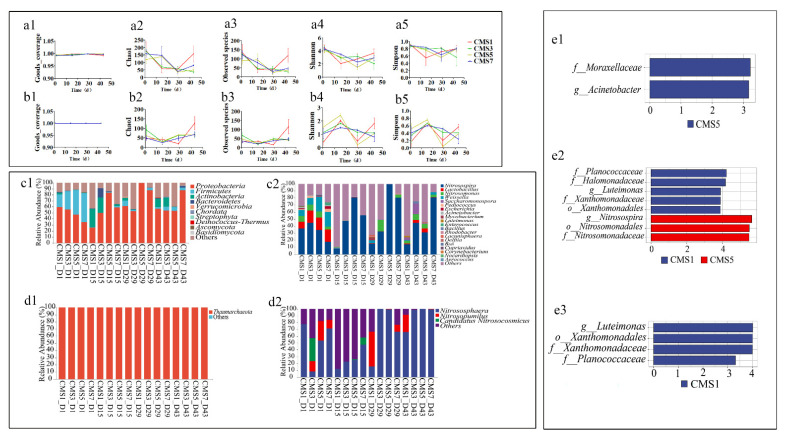
Changes of AOB and AOA community structure in different treatments during the composting. The α diversity of AOB community (**a1**–**a5**) and AOA community (**b1**–**b5**) in different treatments during the composting. The relative abundance of different bacteria at the phylum levels of AOB (**c1**) and AOA (**d1**) and genus levels of AOB (**c2**) and AOA (**d2**) in different treatments during the composting. LDA Effect Size (Lefse) analysis of the biomarkers ((**e1**–**e3**) represent day 15, 29 and 43 for AOB, respectively,) with significant and biological differences from the phylum level to the genus level (LDA score > 3, *p* < 0.05).

AOB and AOA are ubiquitous in various environments and play crucial roles in the nitrogen cycling process [115,116,117]. AOB and AOA also have been found to be common in the composting of various livestock including chicken [118], cow/cattle [4,51,117], sheep [119], pig [120,121]. Based on the Illumina sequencing data, we obtained an average of 56,022 and 113,491 sequence reads per sample ranging from 15,139–140,517 and 40,925–137,869 reads for AOB and AOA, respectively. The alpha diversity indices of AOB and AOA communities in different TF treatments at day1, 15, 29 and 43 of composting were shown in Figure 4(a1–b5), respectively. The Good’s_coverage in every sample was over 0.99, suggesting that the sequencing depth was enough for both AOB and AOA community analysis. The Chao and Observed species indices were significantly higher in the CMS1 treatment than in other treatments (*p* < 0.05) for AOB at mature stage (i.e., day 43), while lower in the CMS1 treatment than in other treatments (*p* < 0.05) for AOA at cooling stage (i.e., day 29). The Shannon and Simpson indices for AOA at the post-thermophilic stage were significantly higher in the CMS5 treatment than the other three treatments, suggesting the inhibition proliferation of AOA communities by high TF treatment through moisture loss or excessive aeration [120]. As mentioned above, the increase of NO_3_^−^-N concentration in the post-thermophilic composting stage may because that AOA were able to oxidize ammonium under thermophilic conditions and high pH [71], or caused by high substrate concentration of NH_4_^+^ to accelerate the nitrification microbial growth [51]. Although it showed relatively higher abundance and diversity at the thermophilic stage of composting in green waste composting [81], microbial activities could be inhibited during thermophilic phase, and the decline in diversity might be attributed to the dominance of some microbial taxa [82]. This could be attributed to the differences in temperature, aeration or moisture caused by different TF during the composting stages. 

The composition and relative abundance of AOB community at phylum and genus levels are shown in Figure 4(c1,c2), respectively. In total, we detected 18 phyla during compost, where Proteobacteria, Firmicutes, Actinobacteria and Bacteroidetes, were the top 4 dominant bacteria, which accounted for 56.16–99.59% of the total sequencing reads. In this study, Proteobacteria played the most dominant role in the whole composting process, accounting for 25.42–99.55%. Interestingly, the relative abundance of Actinomycetes (ranged from 0.27–30.47%) and Bacteroidetes (ranged from 0–15.72%) at the post-thermophilic period reached the highest of 30.47% and 15.72%, respectively. This may indicate that certain Actinomyces and Bacteroidetestes of AOB may survive at high temperature. The relative abundance of dominant phylum species at different composting stages can be affected by different TF, for instance, the relative abundance of Actinomycetes in the high TF treatment was significantly higher than that in the other groups at post-thermophilic period. In total, we detected 141 genera with roles for AOB during the compost, where *Nitrosospira, Lactobacillus, Nitrosomonas, Weissella* and so on were the top 10 dominant bacteria, which accounted for 9.45–99.53% of the total sequencing reads. It was reported that *Nitrosomonas* have frequently been investigated in cattle manure or pig slurry amended soils [122,123], wastewater treating [124] and animal manures composting [4,125]. For AOB, *Nitrosospira* and *Nitrosomonas* usually occupied the first or second dominant position in relative abundance during composting [125]. As previously reported, *Nitrosomonas* was typical ammonia-oxidizing bacteria [126] even dominated all the stages whereas *Nitrosospira* dominated the initial of the composting stage and continually decreased in the maturation stage during cow manure composting [4,125]. However, in present study, *Nitrosospira* played a dominant role in the whole process, especially at the post-thermophilic phase, which reached 8.00%~81.01%; however the proportion of *Nitrosospira* sequences increased at higher temperatures [127]. These findings are in agreement with the previous results that *Nitrosospira* can survive at temperatures of up to 75 °C during composting [128,129]. Interesting, *Nitrosomonas* was almost undetectable during the post-thermophilic phase. These results were in inconsistent with the results that *Nitrosomonas* dominated during all the stages of composting [4,125]. Moreover, the relative abundance of *Nitrosospira* in high TF treatment (i.e., CMS1) was significantly lower than that in the other treatments during post-thermophilic, cooling and maturation stage of the compost, while lower relatively abundant *Nitrosomonas* sequences was detected in too high TF (i.e., CMS1) or too low TF (i.e., CMS7) treatment. These findings suggested that proper availability of oxygen, facilitated by turn or aeration, may be an important regulatory factor for AOB in composts [128].

Microorganisms including bacteria, archaea and fungi played important roles on chicken manure degradation during the composting process [87,130,131]. However, in present study only a total of 3 genera were detected and identified including *Nitrososphaera*, *Nitrosopumilus* and *Candidatus Nitrosocosmicus*, which accounted for 12.18–99.99% of the total sequencing reads (Figure 4(d1,d2) for AOA at phyhum or genus level). These bacteria all belong to Thaumarchaeota phylum. *Nitrosopumilus* and *Nitrososphaera* were the dominant AOA species in animal manure composting [118,120]. In the present study, *Nitrososphaer*a played the most dominant role in the whole composting process, accounting for 8.50–99.94%, which was 12.17–47.21% during post-thermophilic phase, and 15.73–99.74% at the cooling and maturation stage. These findings were in accordance with previously studies that *Nitrososphaera* belong to Thaumarchaeota phylum dominate in compost and can resist high temperatures [118,120]. *Nitrosopumilus* can be detected in the mesophilic, cooling and maturation phase but not in the post-thermophilic stage suggested Nitrosopumilus may prefer to survive or maintain activity at mesophilic stage [132]. Interestingly, the relative abundance of *Nitrososphaera* increased with the decrease of TF at post-thermophilic stage furthermore the relative abundance of *Nitrososphaera* in the high TF treatment was significantly lower than that in the other groups during the post-thermophilic, cooling and maturity periods. These results suggested that the abundance or activity of AOA may be affected by factors such as differing porosity, aeration, or even moisture content [120,133,134]. 

The significant differences among different TF treatments for the AOB community were identified by LEfSe (Figure 4(e1–e3), Supplementary Figure S5(a1–a3), Table S2). The LEfSe of all taxa showed two, eight and four bacterial taxa of AOB were significant different (LDA > 3, *p* < 0.05) among the treatments at the post-thermophilic (i.e., day15), cooling (i.e., day 29) and maturation (i.e., day 43) phases, respectively. Specifically, the dominant taxa were genus as *Acinetobacter, Nitrosospira* and *Luteimona* during post-thermophilic, cooling and maturation phase, respectively. However, LEfSe analysis showed no significant difference among the treatments for AOA community composition. As mentioned above, these results also indicated that the abundance or activity of AOB might be affected by factors such as differing porosity, aeration, or even moisture content [120,133,134]. 

Furthermore, keystone taxa for the microbial communities of the four groups during composting were determined using a random forest model (Supplementary Figure S6(a1,a2,b1,b2)). At the phylum level, Seven taxa were the dominant species occupying the top 10 abundance among the top 10 different important genus for AOB such as *Lactobacillus, Nitrosospira, Weissella, Nitrosomonas, Acinetobacter, Escherichia* and *Corynebacterium*, while *Aerococcus, Muribaculum* and *Pseudomonas* as less abundant species were out of the top 10 abundance genus. In addition, the four groups were consistent with the dominant flora in abundance among species with different importance at the genus level for AOA. 

### 3.5. Key Environmental Factors Shaping Microbial Communities

**Figure 5:**
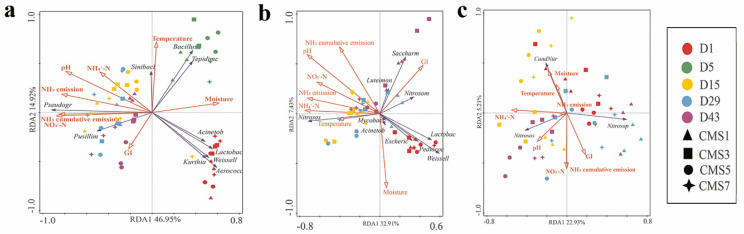
Ordination plots of redundancy analysis for the relationships between environmental factors and bacterial communities at the genus level (**a**), at and between environmental factors and AOB community at the genus level (**b**) and the AOA community at the genus level (**c**) in different treatments during the composting. *Pseudogr* refers to *Pseudogracilibacillus*, *Aerococc* refers to *Aerococcus*, *Lactobac* refers to *Lactobacillus*, *Tepidimi* refers to *Tepidimicrobium*, *Weissell* refers to *Weissella*, *Pusillim* refers to *Pusillimonas*, *Sinibaci* refers to *Sinibacillus*, *Acinetob* refers to *Acinetobacter*, *Nitrosos* refers to *Nitrosospira*, *Nitrosom* refers to *Nitrosomonas*, *Saccharo* refers to *Saccharomonospora*, *Pediococ* refers to *Pediococcus*, *Escheric* refers to *Escherichia*, *Mycobact* refers to *Mycobacterium*, *Luteimon* refers to *Luteimonas*, *Nitrosop* refers to *Nitrosopumilales*, *Nitrosos* refers to *Nitrososphaerales*.

RDA (Redundancy analysis) analysis was applied to study the relationship between microbial succession and physicochemical parameters. Using the 16S rRNA and AOB sequencing data, RDA analysis results at the genus level showed that RDA1 and RDA2 jointly explained 61.87% (Figure 5a) 40.34% (Figure 5b) and 28.14% (Figure 5c) of the total variance, respectively. Further analysis revealed that the temperature, NH_4_^+^-N, NH_4_^+^-N, GI, NH_3_ emission and NH_3_ cumulative emission were the main factors affecting the 16sRNA taxa. *Pusillimonas* and *Pseudogracilibacillus* were positively correlated to NH_3_ emission [103,111]. The pH, moisture, NH_3_ emission and NH_3_ cumulative emission were the main factors affecting the AOB taxa. Interestingly, the moisture content was the only major factor affecting the AOA taxa. This was in agreement with previous study that moisture may affect the amount of dissolved oxygen in the composting [51]. The temperature, pH, NH_4_^+^-N and NO_3_^−^-N had positive effects on the release of NH_3_, while moisture had negative feedback effects on the release of NH_3_. These findings suggested that main environmental variables driving the diversity and structure of AOA and AOB communities were different [135]. In this study the NH_4_^+^-N and NO_3_^−^-N concentration had positive effects on *Nitrososphaera* and *Candidatus Nitrosocosmicus*. This agreed with previous studies, which reported high substrate concentration of NH_4_^+^-N may promote the AOA growth [51,136]. In addition, AOA had a positive relationship with moisture, which supported a previous observation that too high TF accelerated water evaporation and decreased the abundance of ammonia-oxidizing archaea [120].

Further analysis combined with spearman correlation was used to test the correlation between pH, moisture, ammonia release and out numbers of AOA and AOB (Supplementary Tables S3–S5). It was found that pH and NH_3_ release were significantly correlated with the abundance of AOB and AOA (*p* < 0.05). In addition, NH_3_ release was significantly positively correlated with pH and negatively correlated with moisture content (*p* < 0.001) [120]. Specifically, NH_3_ release was significantly negatively correlated with *Lactobacillus, Weissella, Acinetobacter* and positively correlated with *Nitrososphaera* as shown in Supplementary Table S3 during the whole composting (*p* < 0.05). While the NH_3_ release was significantly negatively correlated with *Lactobacillus, Nitrosomonas, Weissella, Acinetobacter* and *Nitrososphaera* on day1 and day15 (shown as Supplementary Table S4), the NH_3_ release was significantly negatively correlated with *Nitrosospira* and positively correlated with *Acinetobacter* on day 29 and day 43 as shown in Supplementary Table S5 (*p* < 0.05). Therefore, in this study AOA and AOB have an important influence on change of NH_3_ emission during composting. *Nitrososphaera* and *Nitrosospira* were significantly negatively correlated with cumulative NH_3_ emission (*p* < 0.05) during the early (on day 1 and day 15) and late (on day29 and day 43) stage of composting, respectively. In addition, it was found that pH, moisture, structure and abundance of microbial community (AOB/AOA) would all affect NH_3_ emission during the composting.

In this study we found that both AOA and AOB have an important influence on the change of NH_3_ emission during composting. *Nitrosospira* and *Nitrososphaera* with high abundance significantly reduced the ammonia emission under turn with suitable frequency during composting of chicken manure. It indicated that we can control the release of NH_3_ through increasing the abundance of ammonia oxidizing bacteria and archaea. The results can provide more novel theoretical support for efficient utilization of livestock and poultry waste.

## 4. Conclusions

This study demonstrated that turning composting materials once every five days (CMS5) had the best effect on the reduction of NH_3_ emission and the compost product quality. *Nitrosospira* and *Nitrososphaera* can convert NH_4_^+^ or NH_3_ to NO_2_^−^ and then NO_3_^−^, resulting in less ammonia emission. The higher relative abundance of ammonia-oxidizing bacteria (*Nitrosospira*) and archaea (*Nitrososphaera*) in the CMS5 treatment could facilitate the aerobic ammonia oxidation and therefore reduce the NH_3_ emission. Too high TF promoted NH_3_ emission and total N loss. Different TF significantly affected the richness and diversity of the bacterial communities during the whole composting stages. Therefore, though only chicken manure and soybean straw were used in the current study, we can choose appropriate aeration to adapt different application options during the process of livestock and poultry waste composting, according to different material composition. Finally, not only the practical use of composting products be achieved but also the economic value of composting products can be improved.

## Data Availability

All data generated or analyzed during this study are included in this published article.

## Supplementary Materials

The following supporting information can be downloaded online. Figure S1: Non-metric multidimensional scaling (NMDS) analysis of bacterial community structure in different turning frequency treatments at various composting stages (a1–a5: represent day1, 5, 15, 29 and 43, respectively). Figure S2: LDA Effect Size (Lefse) analysis of the biomarkers for bacterial community in different treatments. Cladogram showing the biomarkers with significant biological differences from the phylum to genus levels at different composting stages (a1–a5: represent day1, 5, 15, 29 and 43, respectively). Figure S3: Heat map showing the random forest analysis results at the phylum (a1) and genus (a2) levels of bacterial community in different treatments during composting. Figure S4: Non-metric multidimensional scaling (NMDS) analysis of AOB and AOA communities in different turning frequency treatments during various composting stages (a1–a4: represent AOB at day1, 15, 29 and 43, respectively, b1–b4: represent AOA at day1, 15, 29 and 43, respectively). Figure S5: LDA Effect Size (Lefse) analysis of the biomarkers for AOB community in different treatments. Cladogram showing the biomarkers with significant biological differences from the phylum to genus levels at different composting stages (a1–a3: represent day 15, 29 and 43, respectively). Figure S6: Heat map showing the random forest analysis results at the phylum (a1, AOB and b1, AOA) and genus (a2, AOB and b2, AOA) levels of these communities in different treatments during composting. Figure S7: Predicted metabolic pathways involved in composting based on 16S rRNA gene sequencing. Table S1: Numbers of significantly changed phylum/class/order/family/genus identified by the LEfSe analysis based on 16S rRNA gene sequencing. Table S2: Numbers of significantly changed phylum/class/order/family/genus identified by the LEfSe analysis based on AOB gene sequencing. Table S3: Relationships between pH, H_2_O, NH_3_ emission, NH_3_ cumulative emissons and ammonia oxidizing bacteria/archaea of the whole composting (Day 1, 15, 29 and 43). Table S4: Relationships between pH, H_2_O, NH_3_ emission, NH3 cumulative emissons and ammonia oxidizing bacteria/archaea of the mesophilic and thermophilic stage during composting (Day1,15). Table S5: Relationships between pH, H_2_O, NH_3_ emission, NH_3_ cumulative emissons and ammonia oxidizing bacteria/archaea of the cooling and mature stage during composting (Day 29, 43). Table S6: C/N ratio of all treatments during composting.
