# Supplementary material for: Effects of Turning Frequency on Ammonia Emission during the Composting of Chicken Manure and Soybean Straw
Source: Molecules. 2022 Jan 12;27(2):472. doi: 10.3390/molecules27020472 (PMC8777752; doi:10.3390/molecules27020472)
Supplement: Supplementary file 1 [file molecules-27-00472-s001.zip › molecules-1550735-supplementary.pdf]

Supplementary Information

Effects of Turning Frequency on Ammonia Emission during the Composting of Chicken Manure and Soybean Straw

Qianqian Ma [1,2], Yanli Li [1,2], Jianming Xue [3,4], Dengmiao Cheng [5] and Zhaojun Li [1,2,*]

[1] Key Laboratory of Plant Nutrition and Fertilizer, Ministry of Agriculture and Rural Affairs, Institute of Agricultural Resources and Regional Planning, Chinese Academy of Agricultural Sciences, Beijing 100081, China; mqq1027@126.com (Q.M.); liyanli02@caas.cn (Y.L.)
[2] China-New Zealand Joint Laboratory for Soil Molecular Ecology, Institute of Agricultural Resources and Regional Planning, Chinese Academy of Agricultural Sciences, Beijing 100081, China
[3] SCION, Private Bag 29237, Christchurch 8440, New Zealand; jianming.xue@scionresearch.com
[4] College of Biology and the Environment, Nanjing Forestry University, Nanjing 210037, China
[5] Research Center for Eco-Environmental Engineering, Dongguan University of Technology, Dongguan 523808, China; chengdm@dgut.edu.cn
* Correspondence: lizhaojun@caas.cn

Supplementary results

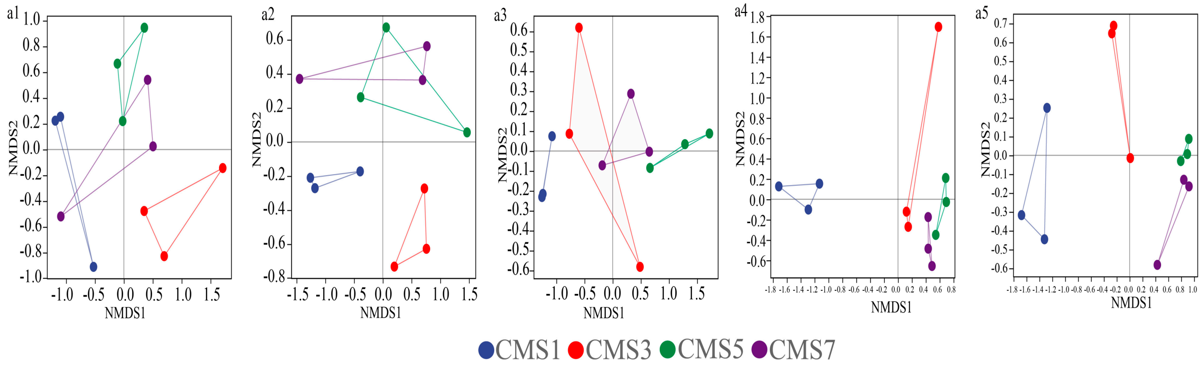

Fig. S1 Non-metric multidimensional scaling (NMDS) analysis of bacterial community structure in different turning frequency treatments at various composting stages (a1-a5: represent day1, 5, 15, 29 and 43, respectively)

NMDS analysis of β-diversity showed that the samples were classified into four groups at post-thermophilic and maturation phase for 16S rRNA (Fig. S1) The CMS1 and CMS3 group could be separated from each other and from the other two groups except for mesophilic and post-thermophilic phase. The CMS1 and CMS5 group could be separated from each other and from the other two groups except for mesophilic and pre-thermophilic phase. Except for the 16S rRNA at mesophilic phase, the stress for all of the treated was less than 0.2.

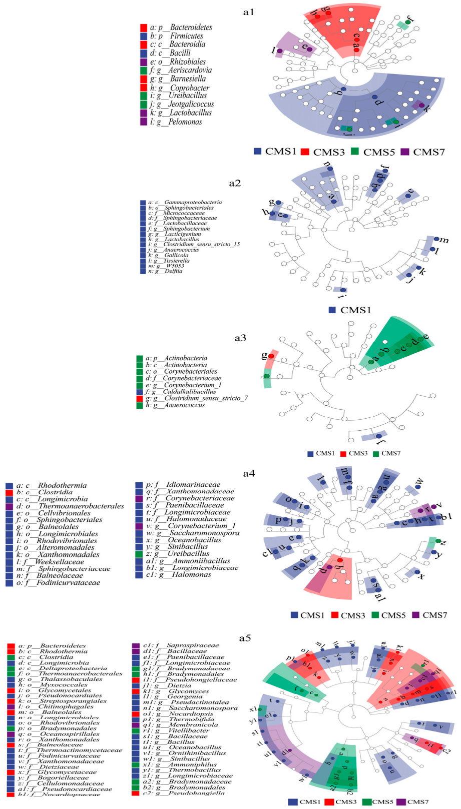

Fig. S2 LDA Effect Size (Lefse) analysis of the biomarkers for bacterial community in different treatments. Cladogram showing the biomarkers with significant biological differences from the phylum to genus levels at different composting stages (a1-a5: represent day1, 5, 15, 29 and 43, respectively)

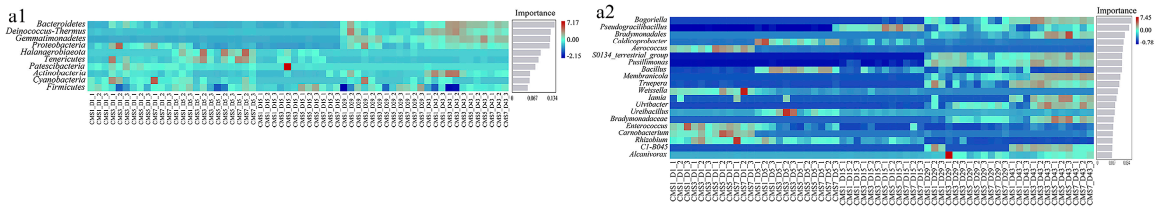

Fig. S3 Heat map showing the random forest analysis results at the phylum (a1) and genus (a2) levels of bacterial community in different treatments during composting

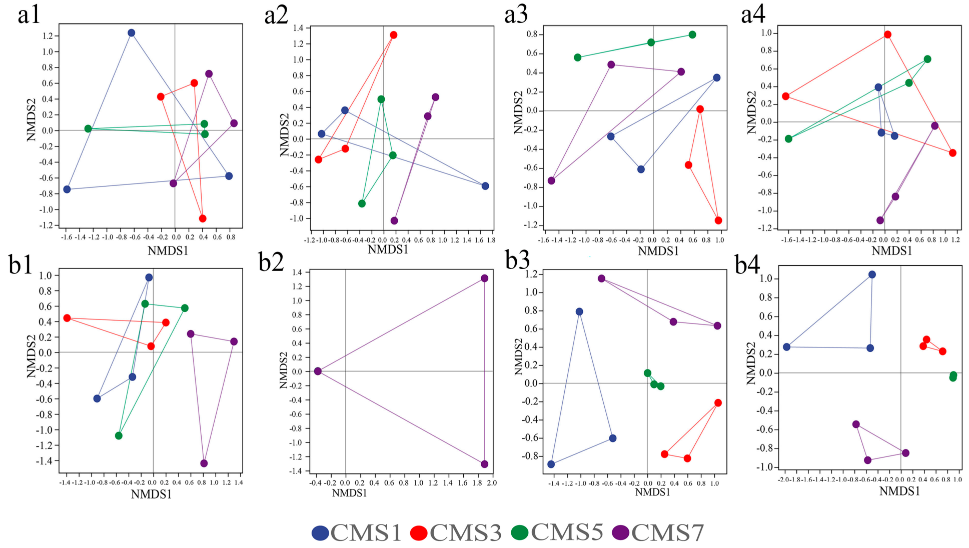

Fig. S4 Non-metric multidimensional scaling (NMDS) analysis of AOB and AOA communities in different turning frequency treatments during various composting stages (a1-a4:represent AOB at day1, 15, 29 and 43, respectively, b1-b4:represent AOA at day1, 15, 29 and 43, respectively)

NMDS analysis of *β*-diversity showed that the samples were classified into four groups at post-thermophilic and maturation phase for AOA (Fig. S4 b1-b4) while AOB was only divided into 4 groups at the post-thermophilic stage (Fig. S4 a1-a4). The CMS1 and CMS3 group could be separated from each other and from the other two groups during cooling stage for AOB. All the treatments could be separated from each other during cooling and mature phase. The stress for all of the treated was less than 0.2.

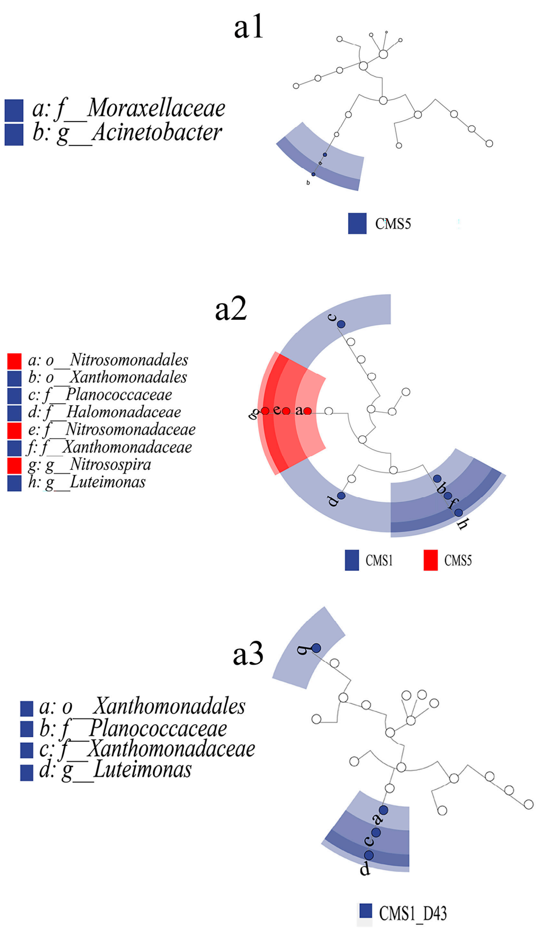

Fig. S5 LDA Effect Size (Lefse) analysis of the biomarkers for AOB community in different treatments. Cladogram showing the biomarkers with significant biological differences from the phylum to genus levels at different composting stages (a1-a3: represent day 15, 29 and 43, respectively)

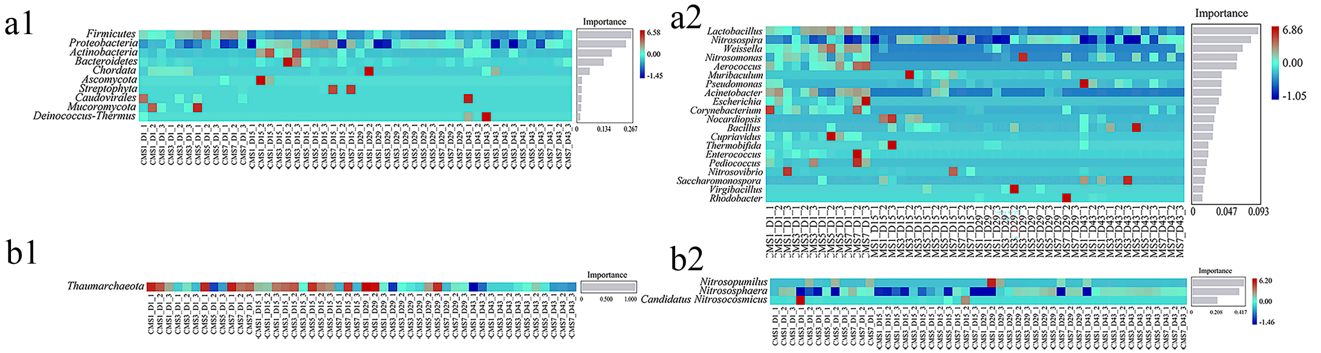

Fig. S6 Heat map showing the random forest analysis results at the phylum (a1, AOB and b1, AOA) and genus (a2, AOB and b2, AOA) levels of these communities in different treatments during composting

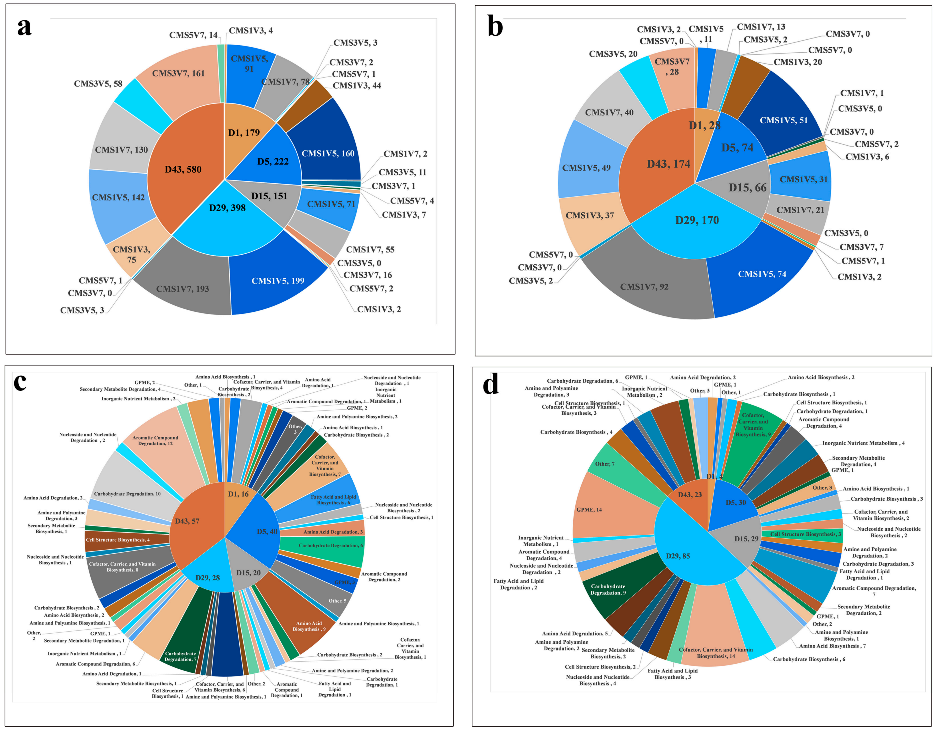

Fig. S7 Predicted metabolic pathways involved in composting based on 16S rRNA gene sequencing

Numbers of significant different metabolic pathways for different treatments (a). Numbers of |FC| greater than 1 for significant different metabolic pathways between different groups (b). The types of metabolic pathways significantly upregulated (c). The types of metabolic pathways significantly downregulated (d).

Note: GPME refers to generation precursor metabolites and energy pathway

According to the MetaCyc database, the abundance of the primary functions identified at days 1, 5, 15, 29 and 43 followed the order of Biosynthesis

(65.20%) > Degradation/Utilization/Assimilation (15.97%) > Generation of Precursor Metabolite and Energy (14.23%), Biosynthesis (64.63%) > Generation of Precursor Metabolite and Energy (16.76%) > Degradation/Utilization/ Assimilation (14.85%), Biosynthesis (67.76%) > Generation of Precursor Metabolite and Energy (16.02%) > Degradation/Utilization/Assimilation (13.16%), Biosynthesis (67.33%) > Generation of Precursor Metabolite and Energy (16.48%) > Degradation/Utilization/ Assimilation (12.87%), Biosynthesis (66.01%) > Generation of Precursor Metabolite and Energy (16.51%) > Degradation/Utilization/Assimilation (13.77%). For the secondary function identification, the dominant pathways included Amino Acid Biosynthesis, Cofactor, Prosthetic Group, Electron Carrier and Vitamin Biosynthesis, and Nucleoside and Nucleotide Biosynthesis (13.01-17.53%).

The number of different metabolic pathways was the largest in the mature stage (i.e. D43), followed by the cooling stage (i.e. D29) and the pre-thermophilic stage (i.e. D5), with 580, 398 and 222, respectively (Fig. S7a). Interestingly, further analysis showed that CMS1 and CMS5 were the groups with the largest number of differences during the mesophilic, pre-thermophilic, post-thermophilic and cooling stages, while CMS3 and CMS7 were the groups with the largest number of differences in the mature stage. This was consistent with the previous physical and chemical index. In the different pathway analysis, CMS1 and CMS7 were the groups with the largest |FC| (fold change) in the mesophilic, cooling and mature stages, while CMS5 and CMS7 were in the mesophilic and mature stages (Fig. S7b). Specifically, as shown in Fig. S7c the highest number of upregulated pathways was Cofactor, Prosthetic Group, Electron Carrier, and Vitamin Biosynthesis between high and low TF groups in mesophilic and pre-thermophilic phase. Aromatic Compound Degradation occurs at both the cooling and maturity stages, while surprisingly, Amino Acid Biosynthesis occurs at the post-thermophilic phase. The highest number of downregulated pathways was amino acid degradation, between high and low TF groups in mesophilic stage. cofactor, prosthetic group, electron carrier, and vitamin biosynthesis occurs at both pre-thermophilic and cooling stages, aromatic compound degradation and carbohydrate degradation occurs at post-thermophilic and the mature phase, respectively (Fig.S7d). It was found that different turning frequencies had the greatest influence on the metabolic pathway in the mature stage, followed by the cooling and pre-thermophilic stage. The different metabolic pathways between the too high (CMS1) and too low (CMS7) TF treatments in each period was similar. This suggested that the effects of too high or too low TF of composting may be similar. The different TF mainly affected Cofactor, Prosthetic Group,

Electron Carrier, and Vitamin Biosynthesis, followed by Amino Acid Degradation, Amino Acid metabolism, Carbohydrate Degradation and Aromatic Compound Degradation.

Table S1 Numbers of significantly changed phylum/class/order/family/genus identified by the LEfSe analysis based on 16S rRNA gene sequencing.

time	Group	[a]N.Clade LDA>3	Taxa	Abundace
	CMS1		Firmicutes	5.97
	CMS1		Bacilli	5.96
	CMS3		Coprobacter	2.51
	CMS3		Barnesiella	2.26
	CMS3		Bacteroidetes	4.64
D1	CMS3	12	Bacteroidia	4.64
	CMS5		Jeotgalicoccus	3.51
	CMS5		Aeriscardovia	3.02
	CMS5		Ureibacillus	2.59
	CMS7		Pelomonas	3.10
	CMS7		Rhizobiales	4.40
	CMS7		Lactobacillus	5.36
	CMS1		Anaerococcus	3.39
	CMS1		Delftia	2.03
	CMS1		Clostridium_sensu_stricto_15	2.95
	CMS1		Gallicola	4.25
	CMS1		Gammaproteobacteria	4.89
	CMS1		Lacticigenium	2.52

	CMS1		Lactobacillaceae	4.46
D5	CMS1	14	Lactobacillus	4.37
	CMS1		Micrococcaceae	2.50
	CMS1		Sphingobacteriaceae	2.10
	CMS1		Sphingobacteriales	2.10
	CMS1		Sphingobacterium	2.10
	CMS1		Tissierella	3.89
	CMS1		W5053	2.88
	CMS1		Caldalkalibacillus	2.42
	CMS3		Clostridium_sensu_stricto_7	2.15
	CMS7		Pseudogracilibacillus	2.46
	CMS7		Anaerococcus	2.99
D15	CMS7	9	Corynebacteriales	4.49
	CMS7		p_Actinobacteria	4.50
	CMS7		Corynebacterium_1	4.49
	CMS7		Corynebacteriaceae	4.49
	CMS7		g_Actinobacteria	4.50
	CMS1		Alteromonadales	2.17
	CMS1		Ammoniibacillus	3.67
	CMS1		Balneolaceae	4.54
	CMS1		Balneolales	4.54
	CMS1		Cellvibrionales	3.49
	CMS1		Fodinicurvataceae	4.11
	CMS1		Halomonadaceae	3.28

	CMS1		Halomonas	3.28
	CMS1		Idiomarinaceae	2.17
	CMS1		Longimicrobia	3.61
	CMS1		g_Longimicrobiaceae	3.61
	CMS1		f_Longimicrobiaceae	3.61
D29	CMS1	29	Longimicrobiales	3.61
	CMS1		Oceanobacillus	4.98
	CMS1		Paenibacillaceae	4.30
	CMS1		Rhodothermia	4.54
	CMS1		Rhodovibrionales	4.11
	CMS1		Saccharomonospora	3.44
	CMS1		Sinibacillus	4.65
	CMS1		Sphingobacteriaceae	4.12
	CMS1		Sphingobacteriales	4.12
	CMS1		Weeksellaceae	3.85
	CMS1		Xanthomonadaceae	4.47
	CMS1		Xanthomonadales	4.47
	CMS3		Clostridia	4.29
	CMS5		Ureibacillus	3.02
	CMS7		Corynebacteriaceae	3.76
	CMS7		Corynebacterium_1	3.76
	CMS7		Thermoanaerobacterales	2.68
	CMS1		Bacillaceae	3.57
	CMS1		Bacillus	4.53

	CMS1		Bogoriellaceae	4.67
	CMS1		Cellulomonadaceae	3.67
	CMS1		Dietzia	3.32
	CMS1		Dietziaceae	3.32
	CMS1		Fodinicurvataceae	4.24
	CMS1		Georgenia	4.66
	CMS1		Longimicrobia	3.73
	CMS1		f_Longimicrobiaceae	3.73
	CMS1		g_Longimicrobiaceae	3.73
	CMS1		Longimicrobiales	3.73
	CMS1		Myxococcales	3.36
	CMS1		Oceanobacillus	5.12
	CMS1		Ornithinibacillus	2.77
	CMS1		Paenibacillaceae	4.07
	CMS1		Pseudactinotalea	3.67
D43	CMS1	55	Pseudonocardiaceae	3.90
	CMS1		Pseudonocardiales	3.90
	CMS1		Rhodovibrionales	4.24
	CMS1		Saccharomonospora	3.89
	CMS1		Sinibacillus	4.60
	CMS1		Thalassobaculales	2.22
	CMS1		Thermoactinomycetaceae	4.23
	CMS1		Thermobacillus	2.46
	CMS1		Thermobifida	4.41
	CMS1		Xanthomonadaceae	4.44

CMS1	Xanthomonadales	4.44
CMS3	Pseudohongiella	2.81
CMS3	Pseudohongiellaceae	2.81
CMS3	Glycomyces	3.60
CMS3	Glycomycetaceae	3.60
CMS3	Glycomycetales	3.60
CMS3	Rhodothermia	4.77
CMS3	Balneolales	4.77
CMS3	Balneolaceae	4.77
CMS3	Bacteroidetes	5.05
CMS3	Streptosporangiales	5.27
CMS3	Nocardiopsis	5.27
CMS3	Nocardiopsaceae	5.27
CMS5	Bradymonadaceae	3.54
CMS5	Vitellibacter	3.50
CMS5	Bradymonadaceae	3.54
CMS5	Thermoanaerobacterales	3.28
CMS5	Ammoniphilus	3.03
CMS5	o_Bradymonadales	4.42
CMS5	f_Bradymonadales	4.42
CMS5	Deltaproteobacteria	4.48
CMS5	g_Bradymonadales	4.48
CMS5	Clostridia	4.62
CMS7	Oceanospirillales	4.55
CMS7	Chitinophagales	4.59

CMS7	Saprospiraceae	4.58
CMS7	Membranicola	4.58
CMS7	Bacillaceae	5.81

[a]Clade LDA > 3: represents the total number of taxa with a LDA > 3; N.Clade: means the number of phylum or class among them.

Table S2 Numbers of significantly changed phylum/class/order/family/genus identified by the LEfSe analysis based on AOB gene sequencing.

Time	Group	[a]N.Clade LDA>3	Taxa	Abundace
D15	CMS5		Acinetobacter	3.46
	CMS5	2	Moraxellaceae	3.55
	CMS1		Xanthomonadales	3.87
	CMS1		Xanthomonadaceae	3.87
D29	CMS1	8	Luteimonas	3.87
	CMS1		Halomonadaceae	3.73
	CMS1		Planococcaceae	4.54
	CMS5		Nitrosomonadaceae	6.00
	CMS5		Nitrosomonadales	6.00
	CMS5		Nitrosospira	6.00
	CMS1		Planococcaceae	3.65
D43	CMS1	4	Xanthomonadaceae	4.38
	CMS1		Xanthomonadales	4.38
	CMS1		Luteimonas	4.37

[a]Clade LDA > 3: represents the total number of taxa with a LDA > 3; N.Clade: means the number of phylum or class among them.

Table S3 Relationships between pH, H_2O, NH_3 emission, NH_3 cumulative emissons and ammonia oxidizing bacteria/archaea of the whole composting (Day1,15,29 and 43).

	pH	H_2O	NH_3 emission	NH_3 cumulative emissons	Nitrosop	Cand Nitr	Nitrososph	Nitrosospi	Lactobac	Nitrosom	Weissell	Acinetob
pH	1											
H_2O	-.433**	1										
NH_3 emission	.664**	0.003	1									
NH_3 cumulative emissons	.591**	-.911**	0.267	1								
Nitrosop	-0.089	-0.108	-0.112	0.05	1							
Cand Nitr	-0.084	0.201	-0.134	-0.223	-0.073	1						
Nitrososph	-0.04	-.351*	-0.245	.345*	-.421**	-0.228	1					
Nitrosospi	0.21	0.021	0.205	0.023	-0.217	0.041	0.16	1				
Lactobac	-.586**	.406**	-.657**	-.530**	0.16	-0.056	-0.039	-.331*	1			
Nitrosom	-0.26	-0.004	-0.197	0.024	-0.157	0.145	.290*	-0.114	0.161	1		
Weissell	-.659**	.447**	-.575**	-.564**	0.253	-0.003	-0.063	-0.266	.764**	0.058	1	
Acinetob	-.549**	.488**	-.527**	-.602**	-0.046	-0.047	0.003	-0.176	.624**	0.073	.510**	1

**: Significant correlation at the level of 0.01 level (bilateral).
*: . Significant correlation at the level of 0.05 (bilateral).

Table S4 Relationships between pH, H_2O, NH_3 emission, NH_3 cumulative emissons and ammonia oxidizing bacteria/archaea of the mesophilic and thermophilic stage during composting (Day1,15).

	pH	H_2O	NH_3 emission	NH_3 cumulative emissons	Nitrosop	CandNitr	Nitrososph	Nitrosospi	Lactobac	Nitrosom	Weissell	Acinetob
pH	1											
H_2O	-.616**	1										
NH_3 emission	.768**	-.774**	1									
NH_3 cumulative emissons	.740**	-.725**	.978**	1								
Nitrosop	-0.223	0.321	-0.342	-0.327	1							
Cand Nitr	0	0.205	-0.141	-0.156	-0.096	1						
Nitrososph	-.483*	0.1	-0.393	-.413*	-0.193	-0.231	1					

	pH	H_2O	NH_3 emission	NH_3 cumulative emissons	Nitrosop	CandNitr	Nitrosoph	Nitrosospi	Lactobac	Nitrosom	Weissell	Acinetob
Nitrosospi	0.228	-.422*	0.131	0.057	-0.038	0.113	-0.093	1				
Lactobac	-.516**	.628**	-.766**	-.733**	.424*	-0.15	0.218	-0.325	1			
Nitrosom	-.739**	.534**	-.663**	-.633**	-0.093	0.364	0.402	-0.076	0.306	1		
Weissell	-.615**	.648**	-.739**	-.704**	.457*	-0.094	0.288	-0.352	.795**	0.321	1	
Acinetob	-.443*	.530**	-.679**	-.652**	-0.056	-0.156	.465*	-0.21	.575**	0.314	0.401	1

**: Significant correlation at the level of 0.01 level (bilateral).
*: . Significant correlation at the level of 0.05 (bilateral).

Table S5 Relationships between pH, H_2O, NH_3 emission, NH_3 cumulative emissons and ammonia oxidizing bacteria/archaea of the cooling and mature stage during composting (Day29,43).

	pH	H_2O	NH_3 emission	NH_3 cumulative emissons	Nitrosop	CandNitr	Nitrososph	Nitrosospi	Lactobac	Nitrosom	Weissell	Acinetob
pH	1											
H_2O	.909**	1										
NH_3 emission	.838**	.781**	1									
NH_3 cumulative emissons	-.726**	-.785**	-.531**	1								
Nitrosop	-0.067	-0.197	0.131	0.191	1							
Cand Nitr	-0.076	-0.043	-0.189	0.107	-0.114	1						
Nitrososph	-0.022	0.158	-0.267	-0.26	-.776**	0.133	1					
Nitrosospi	0.327	.471*	0.306	-.648**	-0.343	-0.004	0.253	1				
Lactobac	-0.37	-0.224	-0.374	0.387	-0.147	0.217	0.211	-.484*	1			
Nitrosom	-0.012	0.051	0.106	0.141	-0.197	0.336	0.243	-0.151	0.297	1		
Weissell	0.035	-0.083	0.042	-0.002	0.22	-0.103	-0.075	-0.126	-0.051	-0.141	1	
Acinetob	0.283	0.155	.406*	-0.091	0.308	-0.051	-0.269	-0.04	-0.022	-0.15	.523**	1

**: Significant correlation at the level of 0.01 level (bilateral).
*: . Significant correlation at the level of 0.05 (bilateral).

Table S6 C/N ratio of all treatments during composting.

	C/N ratio			
Time	CMS1	CMS3	CMS5	CMS7
Day 0	7.57±0.13	7.84±0.24	7.73±0.32	7.34±0.28
Day 1	6.6±0.07a	5.94±0.25b	6.26±0.22b	6.83±0.31a
Day 3	5.1±0.09a	4.58±0.75ab	3.96±0.23b	4.04±0.27b
Day 5	3.78±0.19a	4.02±0.01ab	4.66±0.27c	4.47±0.35bc
Day 7	6.97±0.59a	6.34±0.97a	5.13±0.27b	4.5±0.3b
Day 10	4.91±0.61a	6.17±1.07ab	7.19±0.41b	6.44±0.85b
Day 12	6.02±0.7	6.25±0.57	8.28±0.78	7.4±1.99
Day 15	8.18±0.08	7.2±0.75	6.86±0.97	7.6±0.87
Day 17	10.66±0.25a	9.42±0.09ab	9.07±1.44b	8.67±0.09b
Day 22	9.94±0.6	9.45±1.14	9.47±0.68	10.11±0.46
Day 29	10.18±0.22ab	10.06±0.57ab	10.54±0.05b	9.74±0.28a
Day 37	9.61±0.1a	10.2±0.39ab	10.87±0.62b	9.77±0.61a
Day 43	9.7±0.07	9.8±0.51	9.99±0.11	10.49±0.68
Day 66	9.29±0.3	8.64±0.34	8.81±0.26	8.71±0.52
soybean straw	14.46±0.05			
chicken manure	4.96±0.22			
